# Microbial community dynamics in blood, faeces and oral secretions of neotropical bats in Casanare, Colombia

**DOI:** 10.1038/s41598-024-77090-6

**Published:** 2024-10-28

**Authors:** Nicolas Luna, Luisa Páez-Triana, Angie L. Ramírez, Marina Muñoz, Marcela Goméz, Julián E. Medina, Plutarco Urbano, Karen Barragán, Catalina Ariza, Davinzon Martínez, Carolina Hernández, Luz H. Patiño, Juan David Ramirez

**Affiliations:** 1 Centro de Investigaciones en Microbiología y Biotecnología - UR (CIMBIUR), Facultad de Ciencias Naturales, Universidad del Rosario, Bogotá, Colombia; 2 Instituto de Biotecnología-UN (IBUN), Universidad Nacional de Colombia, Bogotá, Colombia; 3 Grupo de Investigación en Ciencias Básicas (NÚCLEO), Facultad de Ciencias e Ingeniería, Universidad de Boyacá, Tunja, Colombia; 4 Grupo de Investigaciones Biológicas de la Orinoquia, Universidad Internacional del Trópico Americano (Unitrópico), Yopal, Colombia; 5 Centro de Tecnología en Salud (CETESA), Innovaseq SAS, Bogotá, Colombia; 6 Molecular Microbiology Laboratory, Department of Pathology, Molecular and Cell-Based Medicine, Icahn School of Medicine at Mount Sinai, New York, NY USA

**Keywords:** Microbial communities, Bat body fluids, Next-generation sequencing (NGS), Zoonotic diseases, Pathogen transmission, Microbial ecology, Pathogens

## Abstract

Bats are known reservoirs for a wide range of pathogenic microorganisms, including viruses, bacteria, fungi, helminths, and protozoa, which can be transmitted and infect other zoonotic organisms. Various studies have utilised next-generation sequencing (NGS) to describe the pathogens associated with bats. Although most have characterised microbial communities in specific body fluids, few have analysed the composition and diversity of these microbial communities across different body fluids at the individual level. In this study, we employed two next-generation sequencing techniques: amplicon-based sequencing of the V4 hypervariable region of the 16S- and 18S-rRNA genes and viral metagenomics, to describe the prokaryotic, eukaryotic, and viral communities present in blood, faeces, and oral swab samples collected from two genera of bats (*Carollia* and *Phyllostomus*) in the department of Casanare, eastern Colombia. A total of 60 samples corresponding to the three bodily fluids were processed and analysed. The results indicated that the microbial communities across the body fluids were mainly composed of bacteria, fungi, protozoa, and various DNA and RNA viruses, showing a variability of microbial genera and species. The abundances, diversity metrics, and correlations of these microorganisms displayed patterns associated with bat genus and body fluids, suggesting that the ecological characteristics of these microbial communities may be influenced by the ecological and physiological traits of the bats. Additionally, we found similar community compositions of bacteria, some fungal genera, and viruses in the three body fluids, indicating a possible circulation of these microbes within the same bat. This could be due to microbial movement from the gut microbiota to other physiological systems or transmission via blood-feeding vectors. Furthermore, our results revealed the presence of various microbes of public health concern, including *Bartonella* spp., *Mannheimia haemolytica*, *Rhodotorula* spp., Piroplasmida spp., *Toxoplasma gondii*, *Alphacoronavirus* spp., and *Bat circovirus*. The abundance of these pathogenic microbial species across the three bodily fluids suggests potential transmission routes from bats to other organisms, which may contribute to the emergence of zoonotic disease outbreaks. These findings highlight the variability of microorganisms present within the same bat and the different pathogen-host interactions that may regulate the presence and transmission of these zoonotic microbes. Further research is required to elucidate the genomic features, ecological interactions, and biological activities of these microbial communities in bats.

## Introduction

Bats (order Chiroptera) represent one of the most diverse groups of mammals, exhibiting a wide range of adaptations and ecological features. These include varying feeding niches^1^, social structures^2^, and migratory behaviours^3^, all of which are associated with their evolution in diverse ecosystems^4,5^. These adaptations enabled bats to play significant ecological roles, such as seed dispersal^6^., flower pollination^7^, and controllers of insect populations^8^. Additionally, the diversity of evolutionary and ecological adaptations has led to bats being recognised as natural reservoirs and/or hosts for arthropod ectoparasites (e.g., ticks^9^, flies^10^, and mites^11^) and various microorganisms that cause infectious diseases^12^. Among these microorganisms, viruses (e.g., SARS-CoV, Ebola, Henipavirus, and *Lyssavirus*), bacteria (e.g., *Bartonella* and *Borrelia*), protozoa parasites (e.g., Trypanosomatids and *Plasmodium*), and fungi (e.g., *Histoplasma*, *Cryptococcus* and *Paracoccidioides*)^13–15^ stand out, with transmission cycles that involve bats as sources and/or amplifiers of these pathogens.

Anthropogenic activities such as deforestation^16^, habitat fragmentation^17^, and biodiversity loss^18^ are increasing the contact between humans, wildlife, and bats^19^. This increased contact has led to a higher probability of transmitting and spreading various pathogenic microorganisms to new hosts^20–22^. Due to multiple outbreaks of emerging and re-emerging zoonotic diseases associated with bats^23^, including viral^24^, protozoan^23^, bacterial^25^ and fungal^26^ diseases, various studies have focused on analysing the microbes transmitted by these mammals and characterising their transmission and infection cycles^13,15^. During their transmission cycles, the dispersal from bats to other organisms might occur through direct contact with bodily fluids (e.g., saliva, urine, and blood)^27–29^ or through bites^30^. Similarly, this transmission may also occur through indirect mechanisms^27^ such as arthropod vectors^31^, intermediate hosts (e.g., wild or domestic animals)^32^, and the environment, each associated with different transmission routes of pathogens carried by bats^13,27^.

With the increase of several zoonotic diseases associated with bats, various studies have employed molecular characterisation, culture and microscopy-based techniques to analyse the molecular and ecological features of several microorganisms, mainly zoonotic^27,33,34^. Although these techniques identify specific microbial groups and associate bats as wild reservoirs/hosts^35,36^, they hinder the description of the ecology of microbial communities of these mammals. Next-generation sequencing (NGS), particularly amplicon-based sequencing and metagenomics, has enabled the description of the ecology, genomic structure, and evolution of various microbial communities. Using these high-throughput sequencing techniques, different studies have determined the prevalence and diversity of microbial communities within bats^14,37–39^ or characterised the co-occurrence of different microorganisms relevant to public health in various bat body fluids^37,40–43^. Similarly, these methodologies have allowed the description and discovery of various viral communities in bats^15,24,44^, characterising their genomic and evolutionary components^45^, and assessing their potential health impact during transmission to other hosts^38^.

Several studies employing these high-throughput sequencing techniques have analysed various aspects of the microbial communities of bats, providing further insights into the ecology, genomics, and evolutionary relationships of these communities^13–15,46^. In general, the bat microbiome comprises different microorganisms that play various ecological roles as symbionts, commensals, or pathogens^25,47^. Furthermore, the composition, diversity, and ecological interactions of different microorganisms, particularly those with zoonotic potential, may be influenced by ecological and evolutionary traits of bats^48–50^. Specifically, patterns of composition are associated with dietary habits and different anatomical zones or bodily fluids of bats^51–53^, suggesting potential physiological and ecological factors that may regulate the microbiota dynamics in these mammals. Despite the increasing number of studies on microbial ecology in bats, most focus on specific microbial groups or anatomical fluids. Therefore, the ecological characteristics of different microbial communities (prokaryotic, eukaryotic, and viral) in various fluids or anatomical zones of the same bat remain unknown, especially in bats inhabiting endemic areas of infectious diseases.

Understanding the diverse microbial components across various body zones and fluids of bats not only facilitates comprehension of microbial ecology within individual bats^13^, but also enables the description of potential ecological interactions among microorganisms, identification of circulating pathogens, and comprehension of how these microbial communities are structured in endemic areas of infectious diseases. Therefore, in this study, we aim to describe the composition and diversity of various microbial communities (bacteria, fungi, protozoa, and viruses) in blood, faeces, and oral swab samples from two bat genera in the Casanare department (eastern Colombia) using amplicon-based sequencing of 16S- and 18S-rRNA, as well as viral metagenomics.

## Results

### Bat species identification

**Fig. 1:**
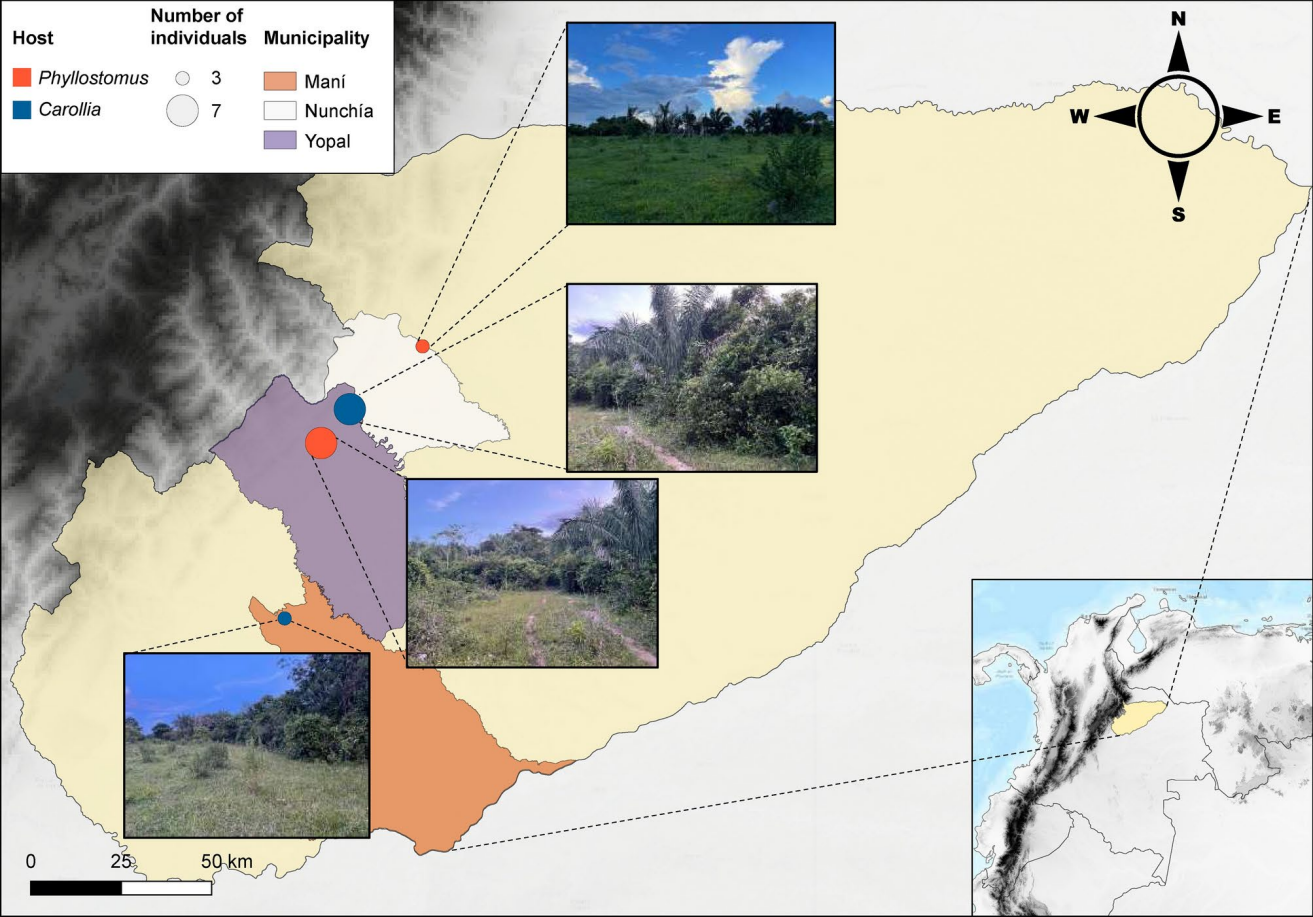
Geographical distribution of the 20 bats collected in the three municipalities of the department of Casanare, Colombia. Each individual was identified by bat species: *Carollia perspicillata* (n = 10), *Phyllostomus hastatus* (n = 9), and *Phyllostomus discolor* (n = 1). Each of the images illustrates the forest edge where the bats were captured. Images were taken by the first author (NL).

We sampled a total of 20 bats across three municipalities in Casanare (Fig. 1), resulting in 60 samples consisting of blood (N = 20), oral swab (N = 20), and faeces (N = 20; Supplementary table 1). By sequencing the mitochondrial 12S gene, we identified three bat species: *Carollia perspicillata* (n = 10), *Phyllostomus hastatus* (n = 9), *and Phyllostomus discolor* (n = 1). These species are distributed throughout the department and possess specific ecological traits: *Phyllostomus* species are mainly classified as omnivorous^54^ and *Carollia* species are categorized as frugivorous^55^.

### Analysis of high throughput sequence data

High-throughput sequencing of 60 bat samples generated an average of 120, 000 raw reads per sample for amplicon-based sequencing (16S- and 18S-rRNA) using Illumina, and 150,800 for viral metagenomics using Oxford Nanopore Technologies (ONT). Rarefaction curves demonstrated that the sequencing depth utilized in amplicon-based sequencing was adequate for assessing the diversity and composition of ASVs present within bat samples (Supplementary Fig. 1). This enabled us to assign 36,466 prokaryotic ASVs and 25, 012 eukaryotic ASVs. After normalization and filtering, we identified 31,834 prokaryotic ASVs and 13,910 eukaryotic ASVs. In ONT, after excluding reads from bats and other microorganisms, we obtained an average of 30, 300 reads per sample, with between 0.05 and 25% of reads classified as viruses.

### Microbial community composition across bat samples

**Fig. 2:**
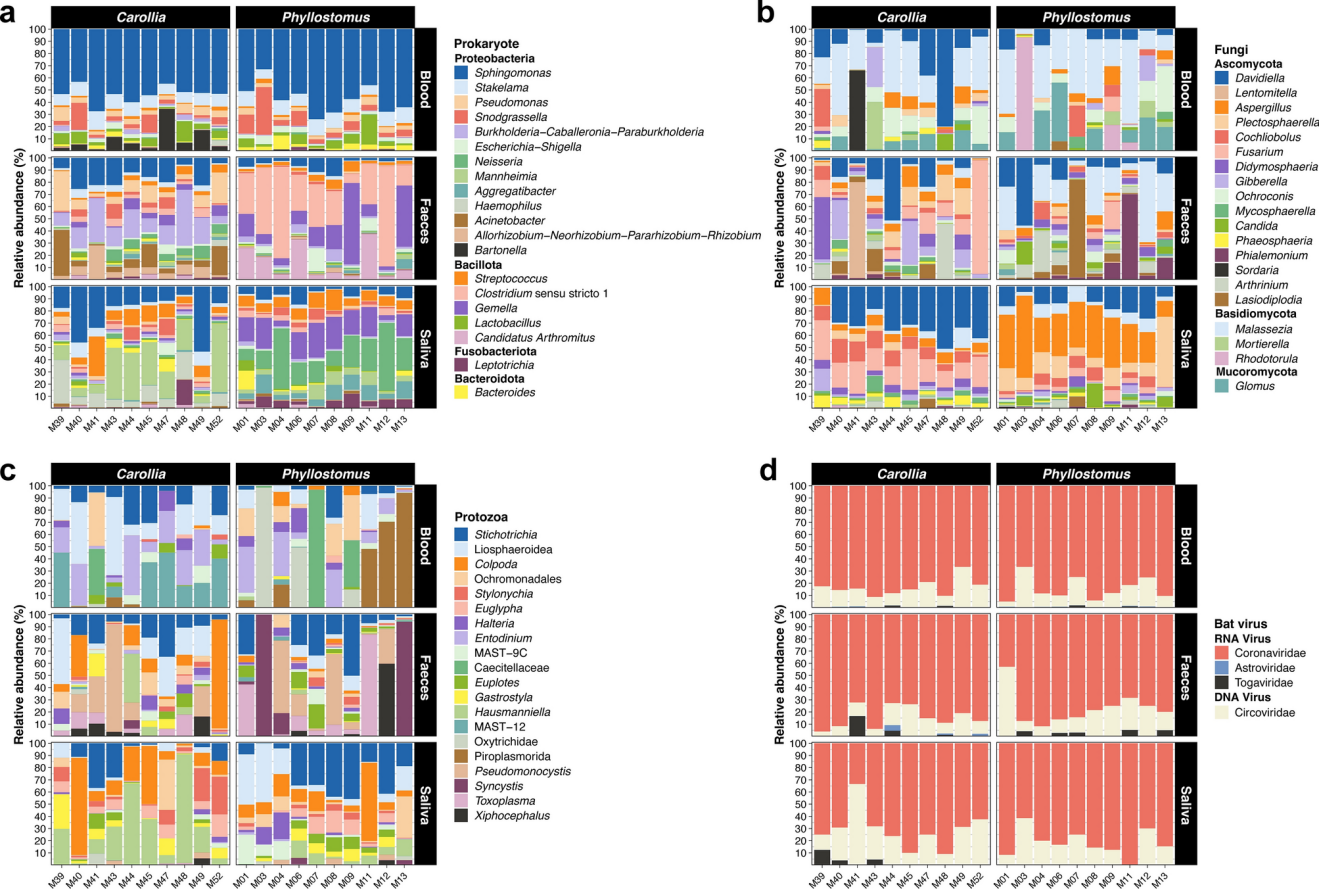
Composition of blood, faeces, and swab microbial communities in Phyllostomidae bats (*Carollia* and *Phyllostomus*). Relative abundances of the most abundant (**a**) bacterial genera, (**b**) fungi genera (**c**), protozoa taxa and (**d**) bat viral families across all samples. For each panel, the stacked bar represents the microbial composition of an individual bat. Images were drawn by the first author (NL).

Analyses of microbial community composition (prokaryotes, fungi, protozoa, and viruses) showed several abundant microbes among the different samples and genera of bats (Fig. 2). Within the prokaryotic communities, most of ASVs belong to bacteria (Supplementary table 2). In these communities, the dominant phyla were Proteobacteria (~ 64.3% across all samples), Bacillota (~ 26.1% across all samples), Bacteroidetes (~ 3.20% across all samples), and Fusobacteriota (1.23% across all samples). At the genus level, we found changes in the composition of the most abundant bacteria across both sample types and bat genera (Fig. 2a; Kruskal–Wallis and U Mann–Whitney tests, *p* < 0.05). In faeces samples, *Clostridium* sensu stricto 1 was the most abundant in *Phyllostomus* (~ 40.9% of relative abundance). In contrast, *Pseudomonas*, *Acinetobacter*, and the *Burkholderia*−*Caballeronia*−*Paraburkholderia* complex were the most dominant in *Carollia* (~ 22.5% of relative abundance). Moreover, in swab samples, *Neisseria*, *Gemella*, and *Streptococcus* were the most abundant genera for *Carollia* (~ 11.7% of relative abundance) and *Mannheimia* and *Haemophilus* for *Phyllostomus* (~ 6.41% of relative abundance). In contrast to this trend, blood samples displayed a different compositional pattern, with *Sphingomonas* being the most abundant in both bat genera (~ 80.6% of relative abundance). On the other hand, we observed some of the genera, such as *Sphingomonas* and *Stakelama*, were present across all bat samples with variations in their relative abundances (Fig. 2a).

In terms of eukaryotes (fungi and protozoa), within fungal communities, Ascomycota (~ 75% across all samples) and Basidiomycota (~ 20% across all samples) were the most abundant phyla, each comprising diverse genera. Similar to bacteria, we observed variations in the composition of these genera based on the sample type and bat genus (Fig. 2b; Kruskal–Wallis and Mann–Whitney U tests, *p* < 0.05). Specifically, *Malassezia* and *Davidiella* were among the most abundant in blood samples for both genera (~ 50% of relative abundance). Moreover, in faeces, *Lasiodiplodia*, *Malassezia*, and *Phialemonium* were the dominant genera in *Phyllostomus* (~ 64.7% of relative abundance), while *Fusarium* and *Didymosphaeria* were abundant in *Carollia* (~ 57.6% of relative abundance). In contrast, we observed a more homogeneous pattern in the abundance of these communities in swab samples. In *Phyllostomus*, *Aspergillus* and *Plectosphaerella* were the most abundant fungal genera (~ 57.4% of relative abundance), whereas *Carollia* exhibited a higher relative abundance of *Davidiella*, *Cochliobolus*, and *Fusarium* (~ 65.7% of relative abundance). Furthermore, we found several fungal genera, such as *Davidiella* and *Malassezia*, present across all bat samples with varying relative abundances (Fig. 2b). In protozoa communities, alveolates were the most abundant group (~ 64.2% across all samples), which comprised various taxonomic groups significantly distributed across the bat samples (Kruskal–Wallis and Mann–Whitney U tests, *p* < 0.05; Fig. 2c). Notably, Piroplasmorida was the most abundant in the blood of *Phyllostomus* (~ 50.5% of relative abundance), while *Entodinium* was the dominant genera in the blood of *Carollia* (~ 21.3% of relative abundance). Furthermore, in faecal samples, *Phyllostomus* showed a high abundance of *Toxoplasma* and *Syncystis* (~ 54.8% of relative abundance), whereas *Pseudomonocystis* was dominant in *Carollia* (~ 22.4% of relative abundance). As for swab samples, *Stichotrichia*, *Colpoda*, and *Hausmanniella* were the most abundant protozoa for both bat genera (~ 80.9% of relative abundance). Finally, we observed variations in the relative abundance of *Stichotrichia* and Liosphaeroidea, which are in all three samples (Fig. 2c).

The viral communities comprised various groups of RNA and DNA viruses (Fig. 2d and Supplementary Fig. 2). For both sample type and bat genus, *Alphacoronavirus*, *Circovirus*, Flaviviridae, and Salasmaviridae were the most abundant viral groups (~ 87.6% of relative abundance). However, in faeces and swab samples, the composition encompassed other abundant viral families, such as Peduoviridae, Alternaviridae, Fusariviridae, Marnaviridae, Virgaviridae, and Caliciviridae (~ 2.10% of relative abundance). We found no variations in the composition and abundances of these viral groups based on the sample and bat genus (Kruskal–Wallis and Mann–Whitney U tests, *p* > 0.05).

### Diversity metrics in bat´s microbiota communities

**Fig. 3:**
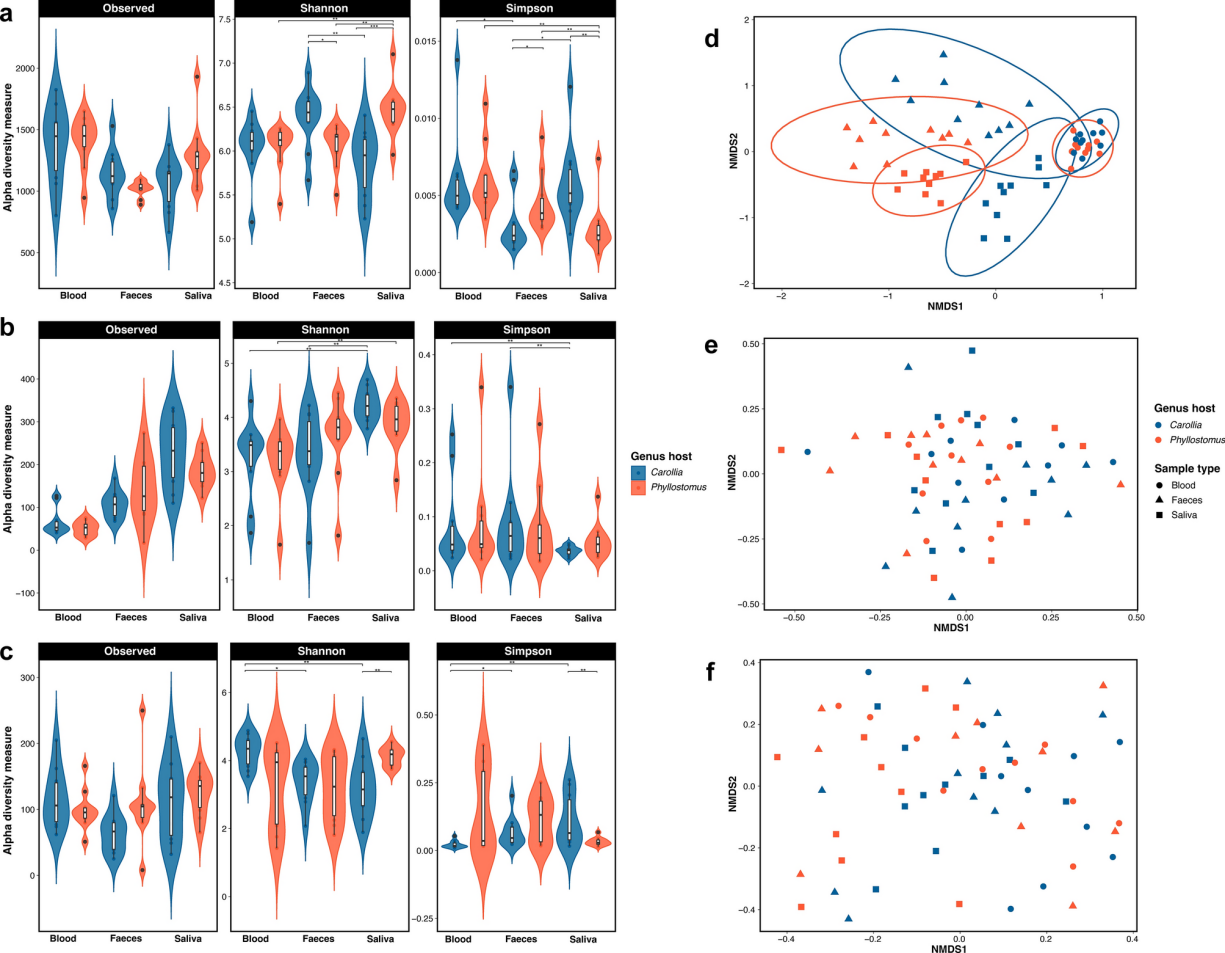
Diversity metrics among blood, faeces, and swab samples from *Carollia* and *Phyllostomus*. Alpha diversity indexes: Number of ASVs (Observed), ASVs diversity (Shannon–Wiener), and ASVs dominance (Simpson) for (**a**) bacteria, (**b**) fungi, (**c**) protozoa communities. Non-metric MultiDimensional Scaling (NMDS) based on dissimilarity (Bray–Curtis distances) for (**d**) bacteria, (**e**) fungi, (**f**) protozoa communities. Significance codes: “*” *p* < 0.05; “**” *p* < 0.01; “***” *p* < 0.001.

At diversity metrics level (Fig. 3 and Supplementary table 3), alpha diversity indices indicate that prokaryotes, fungi, and protozoa communities exhibited a diversity of microorganisms composing their microbial communities across different bat samples and genera (Fig. 3a–c). When assessing these diversity metrics (Shannon–Wiener and Simpson), we found significant differences among bat samples and genera (Kruskal–Wallis and Mann–Whitney U tests, *p* < 0.05). In bacterial communities, the Shannon index was significantly greater in faeces and swab than that blood (Kruskal–Wallis, *p* < 0.05; Fig. 3a). Specifically, within the *Carollia* genus, faeces exhibited higher alpha diversity values, whereas in *Phyllostomus*, it was the swab samples. This pattern of diversity was also observed with Simpson index. Moreover, in fungal communities, swab showed higher diversity values in Shannon and lower values in Simpson. Both indices exhibited significant differences between bat samples (Kruskal–Wallis, *p* < 0.05; Fig. 3b). As for protozoa, there were only differences in *Carollia*, specifically between blood samples and swab and faeces (Kruskal–Wallis, *p* < 0.05; Fig. 3c). Similarly, we observed differences between swab samples for both genera of bats (Mann–Whitney U tests, *p* < 0.05; Fig. 3c).

In terms of beta diversity, Non-metric MultiDimenstional Scaling (NMDS) of Bray–Curtis distance only showed clusters in the bacterial communities (Fig. 3d–f). These clusters exhibited slight separations related to the bat genus and a marked differentiation of the blood microbiota communities from faeces and swab microbiota communities (Fig. 3d). Furthermore, the dissimilarities among bacterial communities in these clusters were associated with the sample type (PERMANOVA test, F = 6.77; *p* = 0.0001), bat genus (PERMANOVA test, F = 14.88; *p* = 0.0002), and their interaction (PERMANOVA test, F = 5.17; *p* = 0.0001). Despite the significance of these factors, we did not find clusters related to geographic location (PERMANOVA test, F = 1.50; *p* = 0.0534).

### Differentially abundant microbes and pathogenic species

The Analysis of Composition of Microbiomes with Bias Correction (ANCOM-BC) among prokaryote and eukaryote communities only identified bacterial ASVs that were differentially abundant among sample types and bat genera (Supplementary Fig. 3). Within these communities, we observed various differential bacteria in the faeces and swab samples of *Carollia* and *Phyllostomus*, respectively. In faeces of *Carollia*, ASVs belonging to *Staphylococcus*, *Pseudomonas*, *Neisseria*, and *Acinetobacter* were among the significantly associated genera. As for swab of *Phyllostomus*, ASVs from *Gemella*, *Aggregatibacter*, *Neisseria*, and *Actinomyces* were part of the differential genera. Among the blood bacterial community, ASVs of *Bartonella* were significantly associated with *Carollia*, while ASV of *Clostridia* UCG−014 was significantly associated with *Phyllostomus*. These findings, along with the composition of the bacterial microbiota, suggest a pattern associated with sample type and bat genus.

**Fig. 4:**
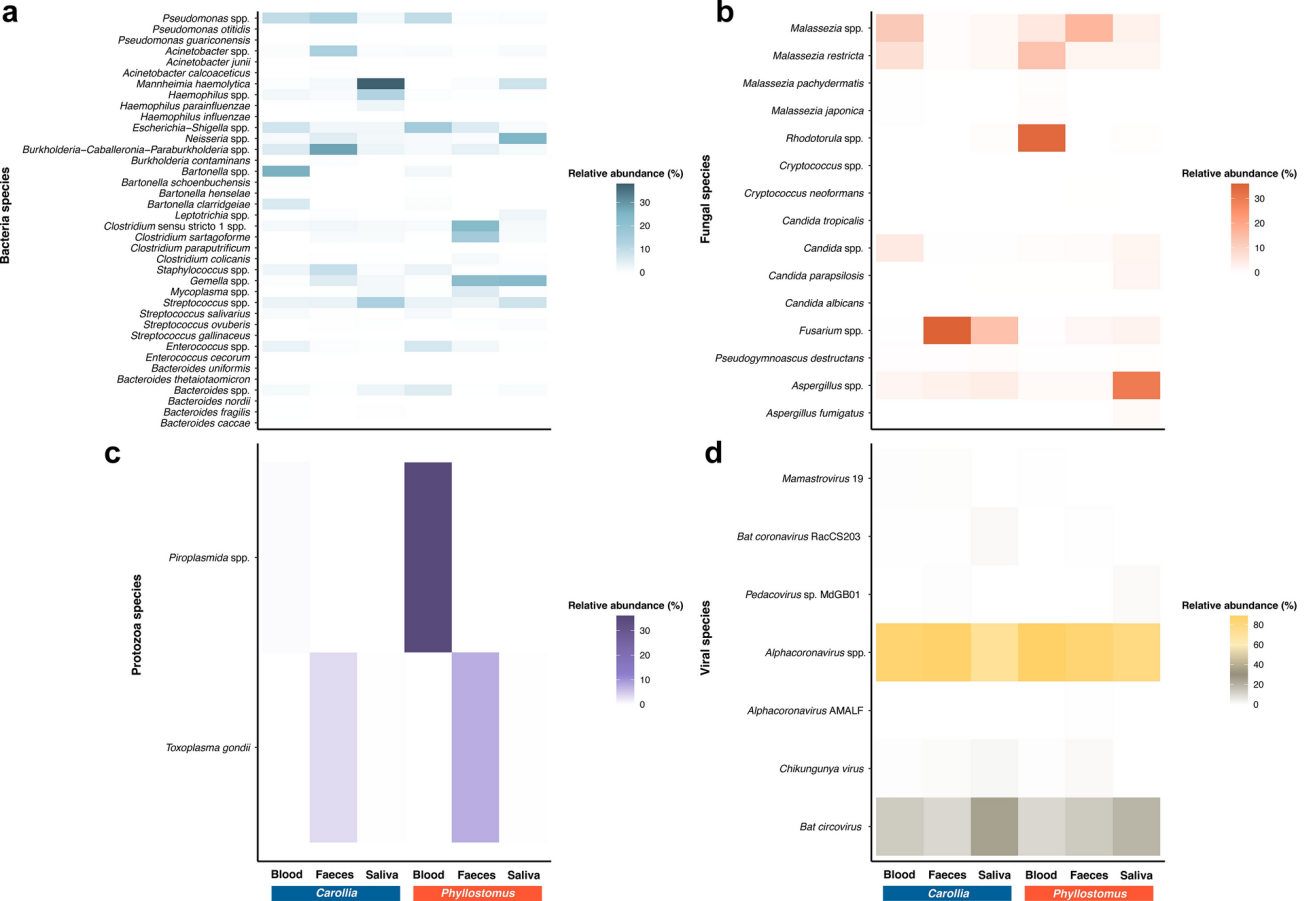
Relative abundances of microbial species pathogenic to mammals. Microbial pathogens in (**a**) bacteria, (**b**) fungal, (**c**) protozoa, and (**d**) viral communities across all bat samples. The heatmap illustrates the mean relative abundances of each identified microbial species in the blood, swab, and faecal samples of the *Carollia* and *Phyllostomus*. Only those species with a relative abundance exceeding 1% are depicted.

Analysing the microbial community compositions and the identification of differentially abundant microbes, we found various taxonomic groups relevant to human and animal health. The taxonomic classification of these genera revealed distinct species whose abundances (> 1%) changed according to sample type and bat genus (Fig. 4). In bacterial communities (Fig. 4a), there were abundances above 15% of *Bartonella* spp., *Burkholderia*−*Caballeronia*−*Paraburkholderia* spp., *Pseudomonas* spp., *Acinetobacter* spp., *Streptococcus* spp. and *Mannheimia haemolytica* in *Carollia* blood, faeces, and swab samples, respectively. Among *Phyllostomus* samples, *Escherichia*−*Shigella* spp., *Clostridium* sensu stricto 1 spp., *Clostridium sartagoforme*, *Gemella* spp., and *Gemella* spp., *Neisseria* spp. were the abundant species in blood, faeces, and swabs, respectively.

Within fungal communities, we observed a similar abundance pattern (Fig. 4b). For *Phyllostomus* samples, there was higher abundance (> 15%) of *Rhodotorula* spp., *Malassezia restricta*, *Malassezia* spp., and *Aspergillus* spp., while *Fusarium* spp. was abundant in *Carollia* samples. Furthermore, in protozoan communities, we found Piroplasmida spp. and *Toxoplasma gondii* as the only species of relevance to human and animal health in blood and faeces samples in both bat genera, respectively (Fig. 4c). In terms of bat viral communities, there were no changes in abundances associated with sample type or bat genus (Fig. 4d). In all three bat samples *Alphacoronavirus* spp. and *Bat Circovirus* were the most dominant viral species (> 20%). Finally, most of the microbial species identified co-occurred in all three types of bat samples (Supplementary Fig. 4). This means that despite variations in their abundances, these microbes can be localized in different physiological systems of bats.

### Correlations of microbial community abundances in bats

**Fig. 5:**
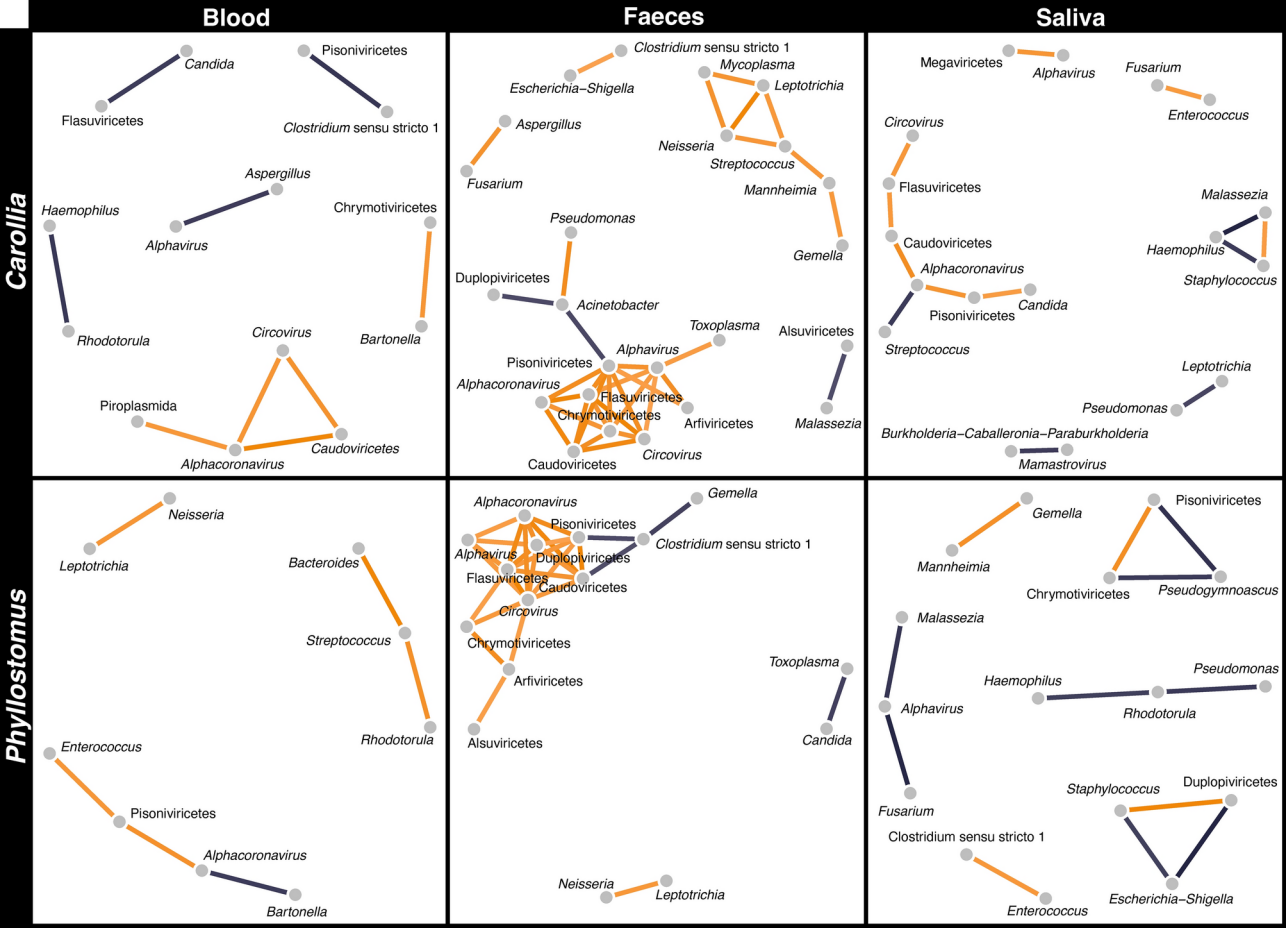
Correlation analysis of the abundances of genera reported as pathogens of mammals and phage classes in blood, swab, and faeces samples. Correlation plot of relative abundances of microbial communities in *Carollia* and *Phyllostomus*. Positive correlations are represented in orange, and negative correlations in purple. Only were considered strong correlations (− 0.75 < *ρ* > 0.75; *p* value < 0.05).

The correlation analysis revealed various inter- and intra-domain interactions across bat samples (Fig. 5). Within faeces samples, there were complex interactions (correlations among more than four taxa), predominantly positive correlations intra-domain, among different viruses (e.g., *Alphacoronavirus*, Pisoniviricetes, *Alphavirus*, Duplopiviricetes, Flasuviricetes, Caudoviricetes *Circovirus*) and bacterial genera (e.g., *Mycoplasma*, *Leptotrichia*, *Neisseria*, *Streptococcus*, *Mannheimia*, *Gemella*). However, these microbes exhibited negative relationships when correlated with other domains (e.g., *Clostridium* sensu stricto 1 with Caudoviricetes and Pisoniviricetes). Moreover, blood and swab showed more simple interactions (correlations between two taxa), mostly inter-domain. For instance, in blood, we observed diverse interactions in *Carollia*, primarily negative, between fungi-bacteria (e.g., *Haemophilus* with *Rhodotorula*), virus-bacteria (e.g., *Clostridium* sensu stricto 1 with Pisoniviricetes), and virus-protozoa (e.g., *Alphacoronavirus* with Piroplasmida). In contrast, in *Phyllostomus*, we found more positive interactions between bacteria (e.g., *Leptotrichia* with *Neisseria*), virus-bacteria (e.g., Pisoniviricetes with *Enterococcus*), and fungi-bacteria (e.g., *Rhodotorula* with *Streptococcus*). In swabs, *Carollia* showed several positive interactions among different viruses and negative interactions between virus-fungi (e.g., Pisoniviricetes with *Candida*) and fungi-bacteria (e.g., *Malassezia* with *Haemophilus*), while in *Phyllostomus*, there were mainly negative interactions between virus-fungi (e.g., *Pseudogymnoascus* with Chrymotiviricetes and Pisoniviricetes) and fungi-bacteria (e.g., *Rhodotorula* with *Haemophilus* and *Pseudomonas*).

## Discussion

The emergence and re-emergence of diseases transmitted by bats have led various studies to analyse and evaluate the microbial communities present in these mammals to understand different ecological, genomic, epidemiological, and evolutionary aspects of these communities^13,14,24,56^. This study characterises the ecology of diverse microbial communities in different body fluids (blood, faeces, and oral secretions) and describes their potential association with the emergence and transmission of pathogenic microbes using amplicon-based sequencing (16S- and 18S-rRNA) and viral metagenomics. Our results highlight three main aspects: the composition of diverse microbial communities, the variation of certain microbial groups across different physiological systems, and the circulation and coexistence of pathogenic microbes relevant for human and animal health.

The compositional and diversity metrics analyses indicate a variability in the microbial taxa present across different bat body fluids (blood, faeces, and oral secretions) (Figs. 2 and 3). These patterns in prokaryotic, fungal, protozoan and viral communities are consistent with the taxonomic profiles and diversity metrics reported in previous studies, which have characterised a variety of microbial communities, mainly Proteobacteria^13,14^, Ascomycota^14^, Basidiomycota^14^ and RNA viruses^15^, in various bat samples (skin^57^, heart^52^, faeces^58,59^, urine^53^, blood^37,43^, kidney^60^, gut^61^ and saliva^53^) using high-throughput sequencing^13,14^. Bats generally host a wide variety of microorganisms, including bacteria, viruses, fungi, and protozoa^13–15^, which have specialised ecological interactions, such as regulating metabolism and nutrient absorption, or influencing pathogen exposure in these mammals^13^. Even though bats host diverse microbial taxa, these microbial diversity and composition features differ from those reported in other mammals^62^. These differences may be attributed to the distinct evolutionary adaptations and ecological traits of bats, such as migration, flight, and dietary niches, which may explain the diversity of microbes hosted by these mammals^50,62,63^.

In our study, most of the variation observed in microbial community composition and diversity metrics is mainly explained by the bat genus. The two genera studied, *Phyllostomus* and *Carollia*, have distinct evolutionary adaptations and ecological behaviours associated with their dietary niches. *Carollia*, primarily categorized as a fruit bat^64^, forages around trees, consuming fruits, seeds, and pollen^55^, whereas *Phyllostomus*, classified as an omnivore^64^, forages at ground level and in tree canopies, consuming insects, small rodents, seeds, and fruits^54^. All these ecological and behavioural differences may explain the observed variations in the composition and diversity of the microbial communities of these genera. Similarly, in another neotropical bat genera, the ecological and behavioural traits, such as dietary strategy, social organization, and reproductive condition, have been reported to determine the diversity, composition, and presence of various microbial communities^40,50,65^. However, due to the lack of specific data on the ecological traits, we are unable to determine which features are causing the observed patterns. Therefore, further research is necessary to thoroughly evaluate the microbial community ecology based on the specific biological traits of these bats.

Another important result is the change in relative abundances and diversity metrics in the microbial communities, mainly in bacterial and fungi, across the samples analysed (Figs. 2, 3 and S3). Each body fluid from the same bat represents a distinct physiological system with specific characteristics that may influence on the ecology of microbial communities. In other mammals, the physiological characteristics of different body fluids have been reported to determine the ecology of microbial communities (composition and diversity), even within the same individual^66^. In bats, previous studies have found that the oral secretions of these mammals contains several enzymatic and biochemical systems, which, in addition to promoting the degradation of different types of food, may regulate the abundance of various microbes^14^. In blood, immunological characteristics have been documented to regulate the abundances and infection of different microorganisms^67^. In faeces, the availability and variation of nutrients and metabolites have been found to determine the ecology of microbial communities^14^. On the other hand, different metagenomic studies suggest that these physiological characteristics influence biological and ecological factors of microbial communities in bats, such as the presence of different microorganisms or ecological interactions with these mammals or other microbes^50,53,68^. Therefore, these functional characteristics between the different physiological systems of bats would be driving the patterns observed in microbial communities. In this sense, along with the biological features of bats, the composition and diversity of microbial communities, especially in bacteria (Fig. 3a, d and Supplementary Fig. 3), may be determined by the physiology, ecology, and evolution of these mammals.

The diversity and co-occurrence of various microbes among body fluids from the same individual (Fig. 2 and Supplementary Fig. 4) highlight the microbe-host interaction, particularly the role of the immune system in these mammals. Given the diversity of microorganisms described in bats, two main hypotheses have been proposed concerning the microbe-host interaction^69^. First, an immunological dampening, wherein bats have evolved suppressive or inactive inflammatory pathways, such as a reduction in the NLRP3 protein family or loss of PYHIN genes, allowing tolerance to various microbes^70,71^. Second, a microbial resistance through constitutive expression of interferons (IFNs), increased heat shock proteins (HSPs), and increased autophagy, favoring mild seroprevalence and premonition states as protective mechanisms against future infections without an exacerbated response^71,72^. Each of these hypotheses has been evaluated using cellular components, as well as genomic and transcriptomic analyses of the immune system in different bat species^12,73,74^. Independent of these hypotheses, the immune features of bats have contributed to a balance between the homeostasis of these mammals and the microbial diversity of pathogens they host, leading them to be considered reservoirs or hosts for various infectious agents.

As for the abundance of microorganisms of the same taxa, particularly bacteria, some fungi, and viruses, across the three bodily fluids (Supplementary Fig. 4), our analyses suggest a potential circulation of various microbes within the same bat. We hypothesise that this circulation could be associated with the movement of these microbes from the gut microbiota to other systems, mediated by the physiological features of the gut epithelium^75^, and/or through transmission via blood-feeding vectors^13,27^. This circulation of different microbes, mainly pathogens, would involve several physiological, ecological, and epidemiological aspects. Firstly, the movement of microorganisms through the different body fluids requires physiological regulation by the bat to maintain homeostasis and microbial activity^12^. Secondly, this circulation could result in changes in the biological activity of the microorganisms, allowing them to colonise and inhabit various bodily fluids^76^. This may lead to alterations in the ecological interactions among different microorganisms and potential modifications in their genomes. Finally, circulation through different fluids may indicate various mechanisms for spreading pathogenic microbes to other hosts, increasing the probability of transmission and the emergence of various zoonotic disease outbreaks^77^. These aspects highlight the importance of future studies on microbe-cell interactions to investigate these hypotheses further, in order to understand the circulation of microorganisms in bats and the potential ecological, physiological, and epidemiological consequences.

In the case of pathogenic species, the analyses using an approach of different body fluids of the same bat revealed several microorganisms of human and animal health concern (Fig. 4). At the individual level (Fig. 2), the patterns in the relative abundances of these pathogenic microbes suggest a potential association between their frequency in the different body fluids and their transmission mechanisms, as well as a coexistence of multiple pathogenic species within the same body fluid. These aspects were observed within the diverse microbial communities analysed. In the case of bacteria (Fig. 4a), the abundances of the pathogenic species were either specific or generalist for the different body fluids. For instance, the higher frequency of *Mannheimia haemolytica* in swab samples may indicate a potential transmission route from bats to other hosts, as this species is primarily known to spread or be transmitted through droplets or saliva^78^. Conversely, several enteropathogenic species, including *Escherichia*-*Shigella* spp., *Enterococcus* spp., *Staphylococcus* spp., *Clostridium* sensu stricto 1 spp., and *Bacteroides* spp., which, in addition to presenting different virulence factors, antibiotic resistance genes^79,80^, and different transmission mechanisms (e.g., faeces, droplets, and saliva)^14,23^, were present in all three body fluids. This suggests not only complexity in the dispersion and infection pathways but also a greater potential for transmitting these bacteria. Therefore, the diverse patterns in the abundance of these pathogenic bacteria in different body fluids would indicate a variety of routes of transmission and infection from bats to other hosts, leading to different outbreaks of associated diseases.

In terms of coexistence, our results highlight a diversity of bacterial species in the same bat or body fluid, suggesting complex dynamics in the ecological, evolutionary, and epidemiological interactions of these microbes. Among the coexistence patterns, we identified several *Bartonella* species, such as *B. schoenbuchensis*, *B. henselae*, and *B. clarridgeiae*, in blood samples (Fig. 4a). These bacteria are known to cause a variety of infectious diseases, including cat scratch disease and trench fever^81^, whose transmission cycles involve bats as wild reservoirs which transmit these species to other organisms via various hematophagous vectors^82,83^. The presence of *B. schoenbuchensis*, *B. henselae*, and *B. clarridgeiae* in bat blood suggests not only the potential transmission of these pathogens to other organisms but also the activity of various associated arthropods. Together with the changes in relative abundances, these findings highlight that the variability and abundance of these microbes in different body fluids may imply various mechanisms of transmission of infectious bacteria and distinct coexistence patterns associated with complex dynamics in the microbial ecology of bats. However, the biological activity of these bacteria needs to be assessed to determine the viability of these microorganisms in each body fluids and clarify the transmission mechanisms of these bacterial species in bats. Additionally, incorporating genomic approaches is necessary to analyse their virulence factors and evolutionary patterns.

Similarly to bacteria, we found coexistence patterns among various opportunistic fungal species such as *Malassezia* spp., *Candida* spp., *Rhodotorula* spp., and *Aspergillus* spp., whose relative abundances showed patterns associated with the different types of samples analysed (Fig. 4b). So far, few studies on fungal communities have described the mechanisms of transmission and dispersal of these fungi in bats or their role in transmission cycles^13,26^. Considering the ecological and immunological features of bats, along with the diversity of fungi documented^13,14^, these mammals may act as hosts that facilitate the transmission of these microorganisms. The frequency of these species in certain fluids may suggest potential pathways for their circulation and dispersal. Moreover, at the individual level, the frequency and prevalence of these species in bat faeces and swab (Fig. 2b) could indicate possible contamination of natural resources (fruits and water resources) by these microorganisms due to the behavioural features of bats. Future studies need to delve into the role of fungi as commensals or pathogens in neotropical bats. Understanding the ecological interactions and potential health impacts of fungal communities in these bats will be crucial for comprehensive insights into their microbial ecology and disease transmission dynamics.

With regard to protozoa, our analysis reveals the presence of *Toxoplasma gondii* and Piroplasmida in bat faeces and blood, respectively (Fig. 4c). These parasites have been documented in various tissues and organs of bats (e.g., heart, muscle, and blood)^84,85^. However, little is known about their ecological associations, the role of bats in the epidemiology of these parasites, and the description of Piroplasmida species in these mammals^86^. From a biological and ecological standpoint, the presence of these microbes in different individuals suggests a potential interaction with arthropod vectors (e.g. ticks) or exposure to natural resources, that may facilitate the transmission of these protozoa^84,85^. Bats might, therefore, act as host in the life cycles of these protozoa, facilitating the dispersion of microorganisms within their ecosystems. To understand their transmission mechanisms and the ecological and evolutionary relationships in bats, future studies should assess the eco-epidemiological features of these parasites using molecular, ecological, and demographic analyses.

Several RNA and DNA viruses were identified in different body fluids at the level of viral communities. These viruses were relevant to public health, including *Alphacoronavirus* sp., *Bat circovirus*, Flaviviridae and Salasmaviridae (Supplementary Figs. 2and 4d). Previous studies have reported that the bat virome in various tissues, organs, and body fluids comprises different viral communities of public health concern^15,24,38^. Such viruses have been identified as the causative agents of different bat-borne zoonotic diseases, including rabies, acute respiratory disease, and respiratory syndrome^15,38^. In neotropical bats, molecular techniques in saliva and faeces samples support the frequency and presence of the different species identified^87,88^. This may indicate a circulation of these viruses in the same individual and suggest possible transmission routes or dispersal mechanisms that could contribute to potential outbreaks of viral diseases. Furthermore, the coexistence of different pathogenic species in each fluid may involve different viral recombination processes, facilitating the emergence of viruses in other hosts^89,90^. Despite describing the composition of viruses using long-read sequencing, it was impossible to assemble complete genomes or segments that could have provided key information on the genomic and evolutionary characteristics of the described communities. Therefore, it is essential to perform whole genome approaches to assess the genomic and evolutionary aspects of the virome of these bats.

The relationships between bacteria, eukaryotes, and viruses in different body fluids are characterised by many interactions, including both synergistic and antagonistic dynamics, as well as competition for energy sources^91–93^. Correlation analyses reveal intricate relationships between diverse groups of pathogenic microbes and phages in bat samples (Fig. 5). These types of correlations in blood, faeces, and swab samples support the hypothesis that physiological and ecological features of bats influence the ecology of distinct microbial communities similar to their dietary niches^94^. Furthermore, the diverse inter- and intra-domain interactions may indicate ecological factors that could influence the prevalence of various pathogenic microorganisms in bats, potentially impacting the transmission and emergence of zoonotic diseases. However, functional interaction analyses are necessary to elucidate the correlations observed in our study.

From an ecological standpoint, the abundance and circulation of various microbes, mainly zoonotic, across different body fluids may suggest a potential dispersal and transmission of these microbes in both sylvatic and urban ecosystems. This is due to the fact that the bats studied bats studied are characterised by sharing living spaces with humans and other animals^95^. Furthermore, the localities in which the bats were captured, which are close to human settlements (Fig. 1), have experienced a reduction in forest cover in recent years due to anthropogenic factors, including livestock farming, fires, and land use^96^. The loss of natural ecosystems and the ability of bats to adapt to different ecosystems would increase the probability of spillover processes occurring from bats to other hosts (humans or domestic animals/livestock), potentially affecting their health through the transmission of these zoonotic microbes^21,22^. Therefore, we emphasise the necessity of further research to understand the frequency of associated zoonotic diseases to these microbes, the dispersal of vectors, the microbial ecology in these reservoirs and other associated hosts, and the transmission efficiency. This is crucial for preventing and mitigating infectious disease outbreaks caused by the transmission of bat-borne microbes.

This study has some limitations. First, the sample size and distribution of bat species sampled, despite revealing patterns associated with individual bat features and body fluids, do not demonstrate potential associations with environmental or geographic variables. Second, there is a lack of bat demographic characteristics (such as sex or age) and information about the periodicity and frequency of various infection disease outbreaks (viral, protozoan, fungal and bacterial). This may offer insights into variations in microbial communities among bats in areas endemic to infectious diseases. Third, although several microorganisms of interest in human and animal health were identified, the biological activity of these communities across the analysed samples remains unknown. Therefore, future studies should not only include a greater number of individuals and ecological-demographic variables of bats, but also analyse the biological activity and genomic structure of these communities to determine the interactions between microorganisms and bats, identify possible sources of transmission and clarify the role of bats during their dispersal cycles. Finally, it is crucial to continue studying the microbial communities of these reservoirs to describe the ecology and genomic features of these infectious agents, thereby establishing mechanisms for the prevention or mitigation of infectious diseases in the future. Despite its limitations, this study is the first to describe the microbial communities (bacteria, fungi, protozoa, and viruses) present in the blood, faeces, and oral secretions of the same individual, as well as the possible role of physiological features and feeding habits in shaping the structure and diversity of these microbes.

## Conclusion

In summary, the microbial communities in different body fluids of the same bat are composed of various microbes, including bacteria, fungi, protozoa, and viruses. The abundance, diversity metrics, and ecological interactions of these communities may vary depending on the ecological traits of the bats and the physiological features of each body fluid. Moreover, analysing different body fluids from the same individual revealed the circulation and coexistence of some members of these microbial communities, primarily pathogens, suggesting potential transmission and dispersal of some these microbes to other hosts. Further studies involving single-cell sequencing, genomics, interaction analyses, and epidemiological data are needed to provide insights into the biological activity, associated metabolites, genomic structure, evolutionary relationships, and ecological interactions of these microbial communities within bats. This study has limitations, including a small sample size and geographical restrictions. Therefore, future research should address these limitations by including other neotropical bat species, particularly those with different dietary habits, such as insectivores, carnivores, and hematophagous bats. Additionally, expanding the description of microbial communities to other reservoirs and bats’ ectoparasites would provide a broader understanding, from a One Health perspective, of the dynamics of these microbes.

## Methods

### Bat sampling collection

During 2022, with the institutional approval of the Ethics Committee of Universidad del Rosario (Resolution No. DVO005 1585-CV1427), different bats were captured using mist nets located at the forest edge in three municipalities (Yopal, Nunchía, and Maní) of the department of Casanare, Colombia (Fig. 1 and Supplementary table 1). The captured individuals were anaesthetised with ketamine. Then, each individual underwent blood extraction by cardiac puncture with an insulin syringe (~ 500 uL), oral secretion collection by oropharyngeal swabbing, and faeces sampling directly from each bat during handling when possible or from the bottom of the retention bag using sterile forceps. All methods were carried out following relevant guidelines and regulations of the ethics committee, we confirm that the study is reported in accordance with ARRIVE guidelines. With the exception of the faecal samples, the samples were transferred to 1 ml of Zymo Shield solution (DNA/RNA Shield, Zymo Research) for nucleic acid preservation. After sampling, all individuals were released once they recovered from anaesthesia.

### Nucleic acid isolation from bat samples

The genetic material was extracted from blood, faeces, and oral swab samples, with half of the volume allocated for DNA extraction and the remaining half for RNA extraction. For DNA extraction, we employed the High Pure PCR Template Preparation kit (Roche Life Science) for blood and swab samples, and the Stool DNA Isolation Kit (Norgen, Biotek Corp.) for faecal samples. The two extraction protocols were conducted following the manufacturer’s instructions, with an elution volume modified to 100 µL. As for the RNA extraction, the Quick-RNA Viral Kit (Zymo Research) was employed with some modifications. Briefly, a preprocessing step was incorporated depending on the type of sample. Faeces samples were incubated in 200 µL of PBS 1X for 12 h and homogenized by disruption with ceramic beads for 5 min at 30 Hz using a TissueLyser II disruptor (Qiagen). Blood and swab samples were mechanically homogenized by pipetting for 30 s to release the biological material. Afterward, the homogenized samples were incubated with 5% v/v of proteinase K (20 mg/ml, Zymo Research) at room temperature for 15 min. Finally, we followed the manufacturer’s instructions with a final elution step of 20 µL of DNase- and RNase-free water, previously preheated to 56 °C.

The integrity, quality and concentration of the extracted nucleic acids were assessed using 1.5% agarose gel electrophoresis and NanoDrop One spectrophotometry. Negative controls with molecular-grade water were included during the extraction process to ensure the absence of cross-contamination between samples.

### Identification of bat species

During sampling, identification to species level was carried out on captured individuals using a traditional taxonomic key. However, due to variations in morphological characters^97^, we conducted species verification of bats through molecular analysis following a protocol described previously^37^. Briefly, we amplified a 215 bp fragment of the mitochondrial 12S gene from blood DNA using primers L1085 (5′-CCCAAACTGGGATTAGATACCCCC-3′) and H1259 (5′-GTTTGCTGAAGATGGCGGCGGTA-3′) in a PCR reaction^98^. The amplification profiles included an initial denaturation at 95 °C for 5 min followed by 35 cycles of denaturation at 95 °C for 30 s, annealing at 57 °C for 15 s and extension at 72 °C for 30 s, and finally an extension at 72 °C for 10 min. The PCR products obtained were purified by ExoSAP-IT^®^ and then subjected for Sanger sequencing. The sequences obtained were analysed with UGENE software and taxonomically assigned by BLAST from the data reported in NCBI^99^.

### 16S- and 18S-rRNA amplicon-based sequencing

To describe prokaryotic and eukaryotic communities, the DNA samples were submitted for amplicon-based sequencing by an independent entity (Novogene, Bioinformatics Technology Co., Ltd, Beijing, China). The sequencing process involved a PCR amplification of the 16S- and 18S-rRNA V4 hypervariable region, using universal primers 515F (5′-GTGCCAGCMGCCGCGGTAA-3′)—806R (5′-GGACTACHVGGGTWTCTAAT-3′) and 528F (5′-GCGGTAATTCCAGCTCCAA-3′)—706R (5′-AATCCRAGAATTTCACCTCT-3′), which enable genus-level identification of prokaryotic^100^ and eukaryotic^101^ communities, respectively. Throughout the PCR reaction, a negative control with molecular-grade water was included as an additional precaution against PCR contaminants. The amplicons were visualized on a 2% agarose gel. Afterward, amplicons for each gene were purified and prepared for library sequencing by end pairing and index adapter ligation. The library was sequenced on a paired-end Illumina platform (Illumina NovaSeq 6000 PE250), generating 250 bp paired-end raw reads with a minimum expected depth of 100 thousand raw reads per sample.

After sequencing, barcodes and adapters were removed from raw sequences using QIIME2 tool^102^. Then, we assessed the quality scores of the sequencing data using FastQC version 0.11.7^103^. This quality control analysis was consolidated using MultiQC version 1.6^104^. Next, we used the DADA2 package^105^ within R software version 4.0.2^106^ to infer amplicon sequence variants (ASVs), unique sequences with 100% identity, from the high-throughput sequencing data. To perform this analysis, we used the default parameters of the microbiome analysis (https://benjjneb.github.io/dada2/tutorial.html). This pipeline filter individual reads based on a Phred score of 30 or higher to minimize misreads, infer the ASVs using the central sample inference algorithm, merge forward and reverse sequences, and remove the chimeric structures^105^. Finally, with DADA2, each ASV was taxonomically assigned by comparing it to the SILVA database version 138.1^107^ for 16S-rRNA and PR2 database version 5.0.1for 18S-rRNA^108^.

### Viral enrichment and oxford nanopre sequencing

RNA samples were used for sequencing and characterizing viral communities in bats. We initially removed ribosomal RNA sequences using the Ribo-Zero Plus rRNA Depletion kit (Illumina), following the manufacturer’s instructions. Subsequently, the samples were enriched using Rapid-SMART9n, which enables the identification of different viruses^109^. Briefly, for cDNA synthesis, 5 µL of RNA depleted, 0.5 µL of the primer RLB-RT9N (5′-TTTTTCGTGCGCCGCTTCAACNNNNNNNNN-3′), and 0.5 µL of dNTPS (New England BioLabs) were mixed and incubated at 65 °C for 5 min, then cooled on ice. The annealed RNA was mixed with 2 μL of SuperScript IV First-strand Buffer, 0.5 μL of DTT, 0.5 μL of RNase OUT, 0.5 μL of RLB TSO (5′-GCTAATCATTGCTTTTTCGTGCGCCGCTTCAACATrGrGrG-3′), and 0.5 μL of SuperScript IV (Invitrogen, Carlsbad). The mixture was incubated at 42 °C for 90 min followed by 10 min at 70 °C to yield the cDNA. Afterward, the cDNA was amplified using 6.25 μL of LongAmp Taq 2X master mix (New England BioLabs), 4.875 μL of Nuclease-free water (NFW), 0.125 μL of RLB primer (5′-TTTTTCGTGCGCCGCTTCA-3′), and 1.25 μL of cDNA. The amplification profiles were as follows: 98 °C for 45 s, 30 cycles of 98 °C for 15 s, 62 °C for 15 s, and 65 °C for 5 min, with a final step at 65 °C for 10 min. During the enrichment process, all samples were quantified to ensure proper execution using the Qubit dsDNA High Sensitivity Assay (Life Technologies) on the Qubit 3.0 instrument (Life Technologies).

The enrichment samples were sequenced by Oxford Nanopore Technologies (ONT). We prepared ONT libraries following the manufacturer’s instructions. Firstly, the NEBNext^®^ Ultra^™^ II End Repair/dA-Tailing Module commercial kit was used to prepare the DNA ends. Then, an unique barcode from the Native Barcoding Kit (EXP-NBD104) was added for each sample using NEBNext^®^ Ultra^™^ II Ligation Module kit. Finally, adapters were ligated using the NEBNext^®^ Quick Ligation Module kit in conjunction with the ligation kit from Oxford Nanopore Technologies (SQK- LSK109). The libraries were loaded into FLO-MIN106 flow cells R9.4.1 on the MinION^™^ MK1C device (ONT) and sequenced using MinKNOW V.3.1.4. program for 72 h.

After sequencing, the raw signal files (FAST5) were processed using ONT Guppy v.6.3.8 with the Super accurate (SUP) model for basecalling, and ONT Guppy barcoder v.6.3.8 for demultiplexing. Next, the ONT sequencing data quality scores were assessed using NanoPack2^110^. Reads aligned to the reference genomes from the GenBank assembly, *Carollia perspicillata*: GCA_004027735.1 and *Phyllostomus hastatus* GCF_019186645.2, were filtered out using Minimap2 software v.2.28.0^111^ and SAMtools v.1.16^112^. An additional filter step was carried out to remove prokaryotic and eukaryotic ribosomal reads through SortMeRNA software v.4.3.6^113^. To analyse viral sequences, the filtered reads were taxonomically assigned with Centrifuge v.1.04^114^ with a minimum length of partial hits (–min-hitlen) of 50 and a k classification parameter of 1. Two custom databases were utilized for taxonomic assignment: the Bat virus database and the Refseq virus database. These databases comprised 995 dereplicated and complete virus genomes/sequences reported in Chiroptera from NCBI Virus for the Bat virus database and 17, 066 dereplicated and complete virus genomes/sequences from RefSeq-NCBI Virus for the Refseq virus database. Centrifuge outputs were processed in *Pavian* package^115^ and RStudio.

### Microbial communities’ analyses

To describe microbial communities, we first filtered out the ASVs corresponding to mitochondria, chloroplast, algae, metazoa, plants, and dinoflagellates from the abundance and taxonomic assignment tables using the R *phyloseq* package v.1.40.0^116^. Before performing follow-up analyses, we rarified our data to equal read depth at the lowest read depth among the samples using the rarefy_even_depth (rngseed = 1) function of *phyloseq* to normalize differences in read depths between samples, which can influence dissimilarity metrics^117^.

In the microbial community features, we identified the 20 most abundant genera in blood, swab, and faeces samples from each individual. This identification was based on the proportion of reads of each ASV or Viral OTU (relative abundance) to the total sample dataset abundance. Differences in the abundances were tested using the non-parametric test U Mann–Whitney and Kruskal–Wallis with Dunn test as post hoc with Benjamini–Hochberg correction. To estimate the diversity of ASVs (alpha (α) diversity), we used the Shannon–Wiener (species diversity) and Simpson (species dominance) indices from the *microbiome* package of R v.1.18.0^118^. Likewise, we evaluated the total prokaryotic and eukaryotic diversity of our samples through rarefaction curve analyses using the iNterpolation and EXTrapolation (*iNext)* v.3.0.0^119,120^ and *ampvis2* v.2.7.31^121^ R packages. The same non-parametric tests were used to evaluate the differences obtained from the alpha diversity indices. Regarding beta (β) diversity, the dissimilarities of the microbiota were assessed and visualized by Non-metric MultiDimenstional Scaling (NMDS) of the *phyloseq* package. This analysis was performed based on Bray–Curtis distances obtained from the relative abundances of each ASVs. Furthermore, we conducted a permutational multivariate analysis of variance test (PERMANOVA) from the *vegan* package v.2.6–2^122^ with 9,999 permutations to assess changes in microbiota communities according to bats’ sample type, genus, and localization.

### Analysis of differential and pathogen microbials

From microbial composition analyses, we identified the genera that are differentially abundant using the Analysis of Composition of Microbiomes with Bias Correction (ANCOM-BC) method from *ANCOMBC* R package v.2.4.0^123,124^, with a FDR-corrected p-value cutoff of 0.05. Subsequently, we classified microbial species of differentially abundant genera and/or genera reported as pathogens of mammals in bats (bacteria, fungi, protozoa, and viruses)^125,126^. Taxonomic assignment for each genus was conducted using Centrifuge software (-k 1 –min-hitlen 150) with a reference database constructed from sequences reported in RefSeq^127^. Finally, the obtained information was cross-referenced and visualized alongside respective abundance values using RStudio. Additionally, we visualized the co-ocurrence of these species within samples of the same bat genus.

### Ecological interactions among bat microbial communities

We examined the correlations among genera reported as pathogens of mammals, along with different classes of phage, to understand the potential ecological relationships of these microbes. For this analysis, the non-parametric Spearman correlation was used, considering only strong correlations with values greater than 0.75 and less than − 0.75^128^ and with statistical significance (*p* < 0.05). The correlation analyses were conducted and visualized in RStudio using the *psych* v.2.4.3^129^ and *ggraph* v.2.2.1^130^ packages, respectively.

## Supplementary Information

Supplementary Information.

## Data Availability

The datasets generated and analysed in this study are available in the ENA repository under project number PRJEB77306.
