# Supplementary material for: Microbial community dynamics in blood, faeces and oral secretions of neotropical bats in Casanare, Colombia
Source: Sci Rep. 2024 Oct 28;14:25808. doi: 10.1038/s41598-024-77090-6 (PMC11519573; doi:10.1038/s41598-024-77090-6)
Supplement: Supplementary file 1 — Supplementary Information. [file 41598_2024_77090_MOESM1_ESM.pdf]

Supplementary information

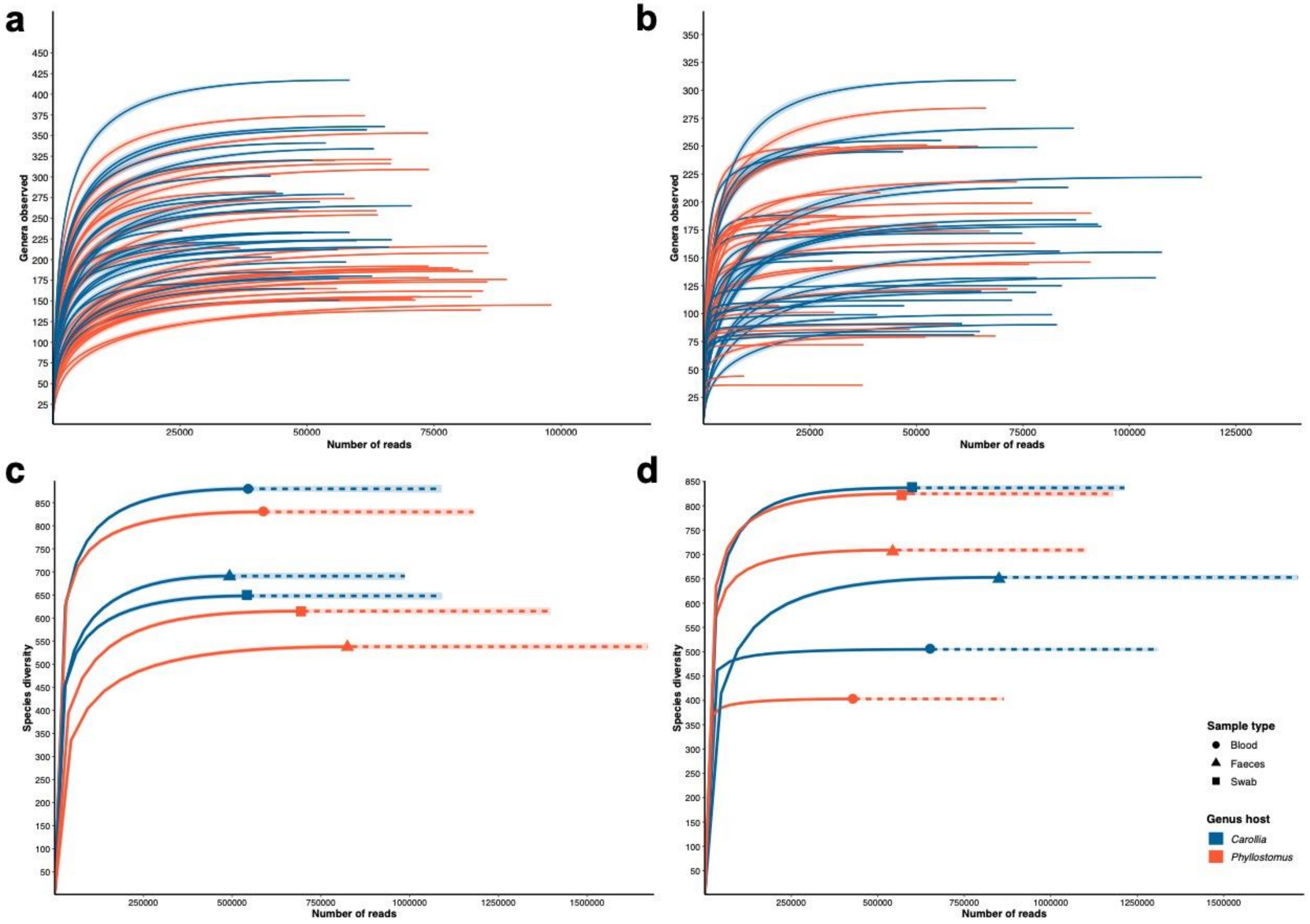

Supplementary figure 1. Rarefaction analysis of prokaryotic and eukaryotic communities. Rarefaction curves of **(a)** prokaryotes and **(b)** eukaryotes in each bat sample. Rarefaction curves of **(c)** prokaryotes and **(d)** eukaryotes by sample type and bat genera.

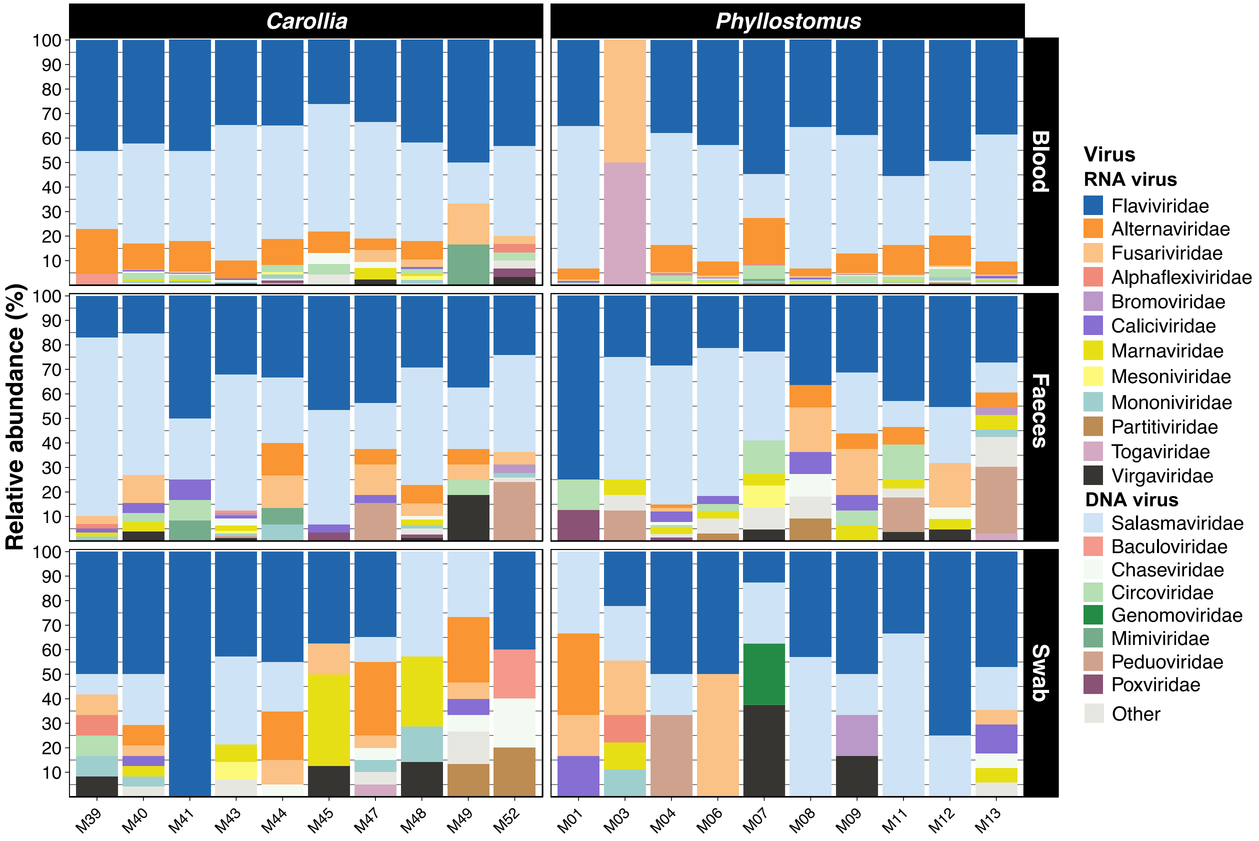

Supplementary figure 2. Composition of viral families in blood, swab, and faecal samples from *Carollia* and *Phyllostomus*. This families were assigned using sequences and reference genomes from RefSeq. For each panel, the stacked bar represents an individual bat.

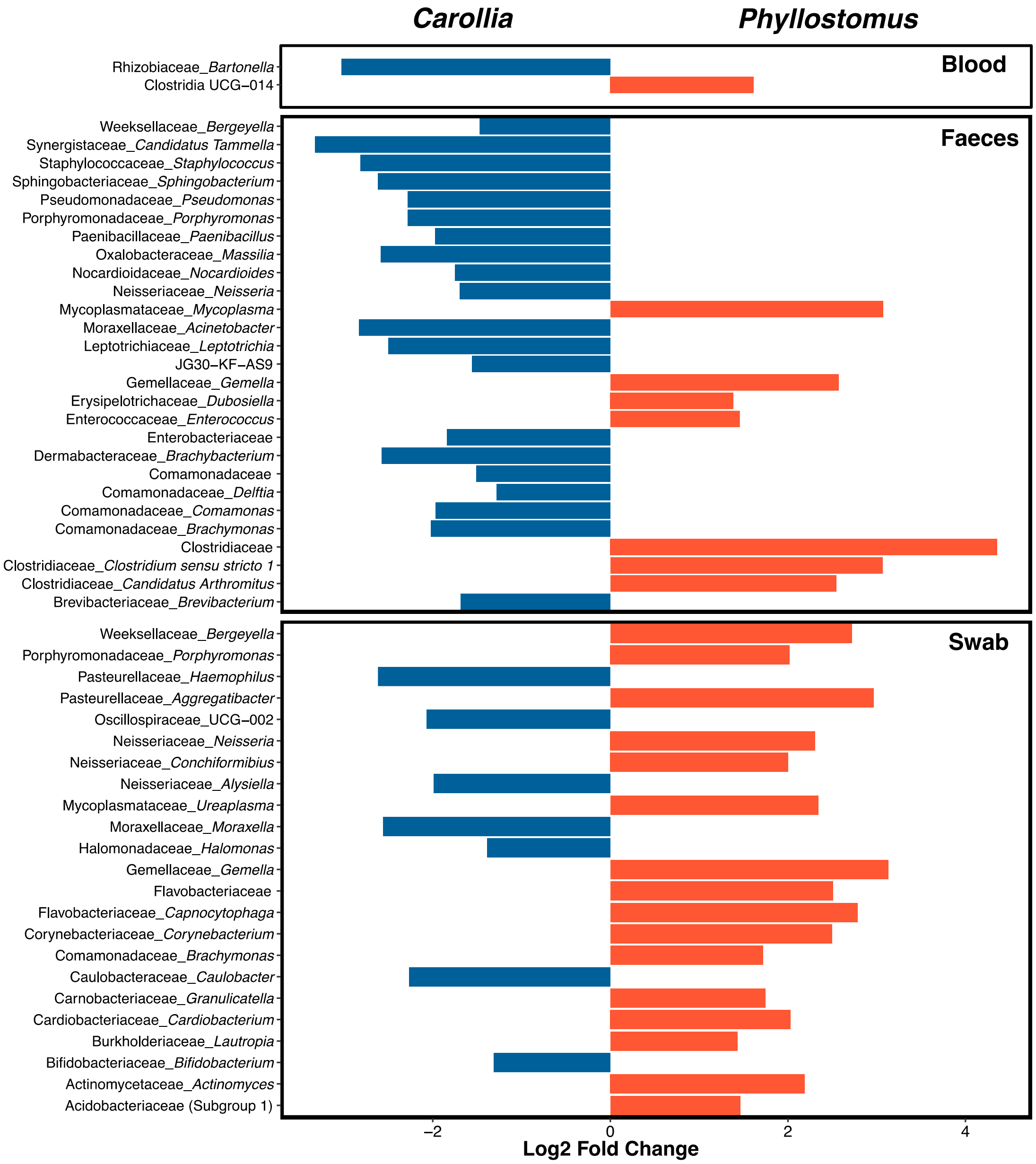

Supplementary figure 3. Analysis of bacterial groups with differential abundance among blood, faeces, and swab samples in *Carollia* and *Phyllostomus*.

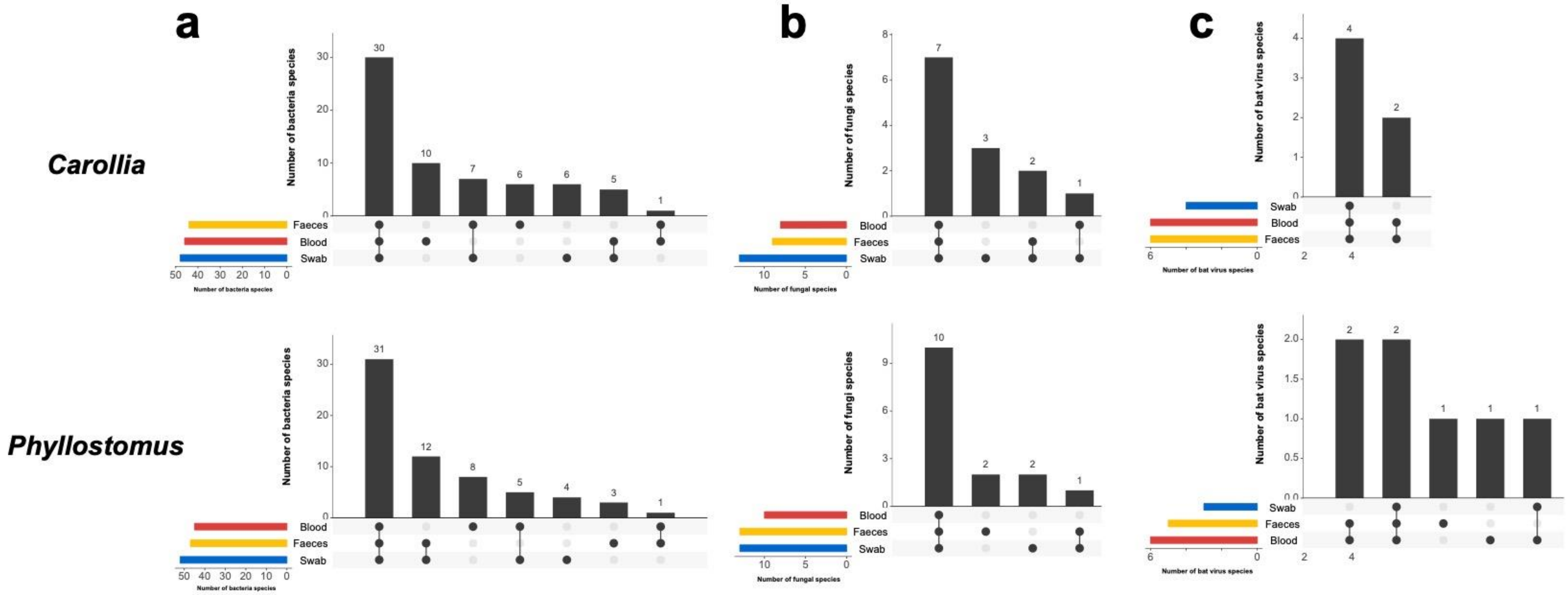

Supplementary figure 4. Co-occurrence of pathogenic microbial species among blood, faeces, and swab samples in *Carollia* and *Phyllostomus*. The figure illustrates the occurrences and co-occurrences of **(a)** bacteria, **(b)** fungi, and **(c)** viral species across body fluids.

Supplementary table 1. Information on each of the bat samples collected in the three municipalities of the department of Casanare eastern Colombia.

Sample ID	Bat	Specie	Sample type	Sex	Municipality	Village	Latitude	Longitude	Date
MH01	M01	*Phyllostomus hastatus*	Swab	NA	Yopal	La Niata	5.399	-72.304	2022-01
MP01	M01	*Phyllostomus hastatus*	Faeces	NA	Yopal	La Niata	5.399	-72.304	2022-01
MS01	M01	*Phyllostomus hastatus*	Blood	NA	Yopal	La Niata	5.399	-72.304	2022-01
MH03	M03	*Phyllostomus hastatus*	Swab	NA	Yopal	La Niata	5.399	-72.304	2022-01
MP03	M03	*Phyllostomus hastatus*	Faeces	NA	Yopal	La Niata	5.399	-72.304	2022-01
MS03	M03	*Phyllostomus hastatus*	Blood	NA	Yopal	La Niata	5.399	-72.304	2022-01
MH04	M04	*Phyllostomus discolor*	Swab	NA	Nunchía	La pradera	5.640	-72.051	2022-01
MP04	M04	*Phyllostomus discolor*	Faeces	NA	Nunchía	La pradera	5.640	-72.051	2022-01
MS04	M04	*Phyllostomus discolor*	Blood	NA	Nunchía	La pradera	5.640	-72.051	2022-01
MH06	M06	*Phyllostomus hastatus*	Swab	NA	Yopal	La Niata	5.399	-72.304	2022-01
MP06	M06	*Phyllostomus hastatus*	Faeces	NA	Yopal	La Niata	5.399	-72.304	2022-01
MS06	M06	*Phyllostomus hastatus*	Blood	NA	Yopal	La Niata	5.399	-72.304	2022-01
MH07	M07	*Phyllostomus hastatus*	Swab	NA	Yopal	La Niata	5.399	-72.304	2022-01
MP07	M07	*Phyllostomus hastatus*	Faeces	NA	Yopal	La Niata	5.399	-72.304	2022-01
MS07	M07	*Phyllostomus hastatus*	Blood	NA	Yopal	La Niata	5.399	-72.304	2022-01
MH08	M08	*Phyllostomus hastatus*	Swab	NA	Yopal	La Niata	5.399	-72.304	2022-01
MP08	M08	*Phyllostomus hastatus*	Faeces	NA	Yopal	La Niata	5.399	-72.304	2022-01
MS08	M08	*Phyllostomus hastatus*	Blood	NA	Yopal	La Niata	5.399	-72.304	2022-01
MH09	M09	*Phyllostomus hastatus*	Swab	NA	Yopal	La Niata	5.399	-72.304	2022-01
MP09	M09	*Phyllostomus hastatus*	Faeces	NA	Yopal	La Niata	5.399	-72.304	2022-01
MS09	M09	*Phyllostomus hastatus*	Blood	NA	Yopal	La Niata	5.399	-72.304	2022-01
MH11	M11	*Phyllostomus hastatus*	Swab	NA	Yopal	La Niata	5.399	-72.304	2022-01
MP11	M11	*Phyllostomus hastatus*	Faeces	NA	Yopal	La Niata	5.399	-72.304	2022-01
MS11	M11	*Phyllostomus hastatus*	Blood	NA	Yopal	La Niata	5.399	-72.304	2022-01
MH12	M12	*Phyllostomus discolor*	Swab	NA	Nunchía	La pradera	5.640	-72.051	2022-01
MP12	M12	*Phyllostomus discolor*	Faeces	NA	Nunchía	La pradera	5.640	-72.051	2022-01
MS12	M12	*Phyllostomus discolor*	Blood	NA	Nunchía	La pradera	5.640	-72.051	2022-01
MH05	M13	*Phyllostomus discolor*	Swab	NA	Nunchía	La pradera	5.640	-72.051	2022-01
MP05	M13	*Phyllostomus discolor*	Faeces	NA	Nunchía	La pradera	5.640	-72.051	2022-01
MS05	M13	*Phyllostomus discolor*	Blood	NA	Nunchía	La pradera	5.640	-72.051	2022-01
MH22	M39	*Carollia perspicillata*	Swab	Male	Maní	El viso	4.962	-72.395	2022-02
MP22	M39	*Carollia perspicillata*	Faeces	Male	Maní	El viso	4.962	-72.395	2022-02
MS22	M39	*Carollia perspicillata*	Blood	Male	Maní	El viso	4.962	-72.395	2022-02
MH13	M40	*Carollia perspicillata*	Swab	Female	Maní	El viso	4.962	-72.395	2022-02
MP13	M40	*Carollia perspicillata*	Faeces	Female	Maní	El viso	4.962	-72.395	2022-02
MS13	M40	*Carollia perspicillata*	Blood	Female	Maní	El viso	4.962	-72.395	2022-02
MH14	M41	*Carollia perspicillata*	Swab	Female	Maní	El viso	4.962	-72.395	2022-02
MP14	M41	*Carollia perspicillata*	Faeces	Female	Maní	El viso	4.962	-72.395	2022-02
MS14	M41	*Carollia perspicillata*	Blood	Female	Maní	El viso	4.962	-72.395	2022-02
MH16	M43	*Carollia perspicillata*	Swab	Male	Yopal	La chaparrera	5.483	-72.233	2022-02
MP16	M43	*Carollia perspicillata*	Faeces	Male	Yopal	La chaparrera	5.483	-72.233	2022-02
MS16	M43	*Carollia perspicillata*	Blood	Male	Yopal	La chaparrera	5.483	-72.233	2022-02
MH17	M44	*Carollia perspicillata*	Swab	Male	Yopal	La chaparrera	5.483	-72.233	2022-02
MP17	M44	*Carollia perspicillata*	Faeces	Male	Yopal	La chaparrera	5.483	-72.233	2022-02
MS17	M44	*Carollia perspicillata*	Blood	Male	Yopal	La chaparrera	5.483	-72.233	2022-02
MH18	M45	*Carollia perspicillata*	Swab	Female	Yopal	La chaparrera	5.483	-72.233	2022-02
MP18	M45	*Carollia perspicillata*	Faeces	Female	Yopal	La chaparrera	5.483	-72.233	2022-02
MS18	M45	*Carollia perspicillata*	Blood	Female	Yopal	La chaparrera	5.483	-72.233	2022-02
MH19	M47	*Carollia perspicillata*	Swab	Male	Yopal	La chaparrera	5.483	-72.233	2022-02
MP19	M47	*Carollia perspicillata*	Faeces	Male	Yopal	La chaparrera	5.483	-72.233	2022-02
MS19	M47	*Carollia perspicillata*	Blood	Male	Yopal	La chaparrera	5.483	-72.233	2022-02
MH23	M48	*Carollia perspicillata*	Swab	Male	Yopal	La chaparrera	5.483	-72.233	2022-02
MP23	M48	*Carollia perspicillata*	Faeces	Male	Yopal	La chaparrera	5.483	-72.233	2022-02
MS23	M48	*Carollia perspicillata*	Blood	Male	Yopal	La chaparrera	5.483	-72.233	2022-02
MH24	M49	*Carollia perspicillata*	Swab	Male	Yopal	La chaparrera	5.483	-72.233	2022-02
MP24	M49	*Carollia perspicillata*	Faeces	Male	Yopal	La chaparrera	5.483	-72.233	2022-02
MS24	M49	*Carollia perspicillata*	Blood	Male	Yopal	La chaparrera	5.483	-72.233	2022-02
MH21	M52	*Carollia perspicillata*	Swab	Male	Yopal	La chaparrera	5.483	-72.233	2022-02
MP21	M52	*Carollia perspicillata*	Faeces	Male	Yopal	La chaparrera	5.483	-72.233	2022-02
MS21	M52	*Carollia perspicillata*	Blood	Male	Yopal	La chaparrera	5.483	-72.233	2022-02

Supplementary table 2. Count of the number of reads, ASVs, phyla and genus found in archaea and bacteria.

Kingdom	Number of reads	Number of ASVs	Number of phyla	Number of genera
Archea	580	76	5	10
Bacteria	2146320	31745	65	1341

Supplementary table 3. Alpha diversity indices in prokaryotic, fungal and protozoan communities in blood, faeces and swab of *Carollia* and *Phyllostomus*.

**Prokaryotic communities**						
**Alpha diversity index**	*Carollia*			*Phyllostomus*		
	**Blood**	**Faeces**	**Swab**	**Blood**	**Faeces**	**Swab**
Observed ASVs	1384.4 ± 208.61	1144.3 ± 189.82	1058.7 ± 210.07	1408.6 ± 208.61	1018.6 ± 65.281	1301.4 ± 256.37
Shannon diversity index	6.0472 ± 0.3434	6.3788 ± 0.3445	5.8673 ± 0.3835	6.0554 ± 0.2565	6.0582 ± 0.2417	6.4711 ± 0.2884
Simpson diversity index	0.0059 ± 0.0029	0.0031 ± 0.0017	0.0058 ± 0.0026	0.0060 ± 0.0022	0.0045 ± 0.0019	0.0029 ± 0.0017

**Fungi communities**						
**Alpha diversity index**	*Carollia*			*Phyllostomus*		
	**Blood**	**Faeces**	**Swab**	**Blood**	**Faeces**	**Swab**
Observed ASVs	66.1 ± 208.61	106.9 ± 31.061	228.2 ± 78.851	50.8 ± 16.498	142 ± 74.992	184.9 ± 39.017
Shannon diversity index	3.2461 ± 0.7262	3.3444 ± 0.7419	4.2346 ± 0.2920	3.2150 ± 0.6304	3.6346 ± 0.7594	3.8883 ± 0.4373
Simpson diversity index	0.0844 ± 0.0807	0.0903 ± 0.0936	0.0367 ± 0.0087	0.0864 ± 0.0940	0.0818 ± 0.0787	0.0554 ± 0.0327

**Protozoa communities**						
**Alpha diversity index**	*Carollia*			*Phyllostomus*		
	**Blood**	**Faeces**	**Swab**	**Blood**	**Faeces**	**Swab**
Observed ASVs	116.2 ± 44.551	63.4 ± 29.247	112 ± 57.695	99.3 ± 30.273	107.5 ± 59.810	124.4 ± 32.363
Shannon diversity index	4.2651 ± 0.4616	3.3687 ± 0.6223	3.1835 ± 0.8445	3.2657 ± 1.2233	3.2152 ± 0.9566	4.1189 ± 0.2719
Simpson diversity index	0.0222 ± 0.0138	0.0679 ± 0.0548	0.1108 ± 0.0932	0.1440 ± 0.1578	0.1158 ± 0.0850	0.0330 ± 0.0154
